# A Detailed Review Study on Potential Effects of Microplastics and Additives of Concern on Human Health

**DOI:** 10.3390/ijerph17041212

**Published:** 2020-02-13

**Authors:** Claudia Campanale, Carmine Massarelli, Ilaria Savino, Vito Locaputo, Vito Felice Uricchio

**Affiliations:** Water Research Institute-Italian National Research Council (IRSA-CNR), Bari, BA, Italy; carmine.massarelli@ba.irsa.cnr.it (C.M.); ilaria.savino@ba.irsa.cnr.it (I.S.); vito.locaputo@ba.irsa.cnr.it (V.L.); vito.uricchio@ba.irsa.cnr.it (V.F.U.)

**Keywords:** microplastics, additives, human health, nanoplastics

## Abstract

The distribution and abundance of microplastics into the world are so extensive that many scientists use them as key indicators of the recent and contemporary period defining a new historical epoch: The Plasticene. However, the implications of microplastics are not yet thoroughly understood. There is considerable complexity involved to understand their impact due to different physical–chemical properties that make microplastics multifaceted stressors. If, on the one hand, microplastics carry toxic chemicals in the ecosystems, thus serving as vectors of transport, they are themselves, on the other hand, a cocktail of hazardous chemicals that are added voluntarily during their production as additives to increase polymer properties and prolong their life. To date, there is a considerable lack of knowledge on the major additives of concern that are used in the plastic industry, on their fate once microplastics dispose into the environment, and on their consequent effects on human health when associated with micro and nanoplastics. The present study emphasizes the most toxic and dangerous chemical substances that are contained in all plastic products to describe the effects and implications of these hazardous chemicals on human health, providing a detailed overview of studies that have investigated their abundance on microplastics. In the present work, we conducted a capillary review of the literature on micro and nanoplastic exposure pathways and their potential risk to human health to summarize current knowledge with the intention of better focus future research in this area and fill knowledge gaps.

## 1. The Plasticene

In the last 70 years, we have abetted an increasing growth in the worldwide plastics production, which has consequently spread into the environment to such a point that we can say to live in a plastic world [1,2]. These synthetic polymers are environmental pollutants themselves and act as vectors of transport of various kind of chemicals [3], but they are also considered valid indicators of the recent and contemporary period, generally after the middle of the 20th century [4].

Nowadays, microplastic particles have been ubiquitously detected in a broad range of shapes, polymers, sizes and concentrations in the environments of marine water, freshwater [5], agroecosystems [6], atmosphere [7], food [8] and drinking-water [9], biota [10], and other remote locations [11].

They can be as thin as small veils and be carried away by the wind from miles away, or they can be hard and compact like rocks [12].

Their worldwide distribution is so vast that many scientists use it as a key geological indicator of the Anthropocene [4].

Plastic materials can be used as stratigraphic markers in the archaeological field by considering them as recent and precise indicators of earth deposits.

Some authors identify the period from 1945 onwards as a moment of a significant increase in plastics deposition, to the point that they have used this stratigraphic marker as an excellent indicator [13].

**Figure 1:**
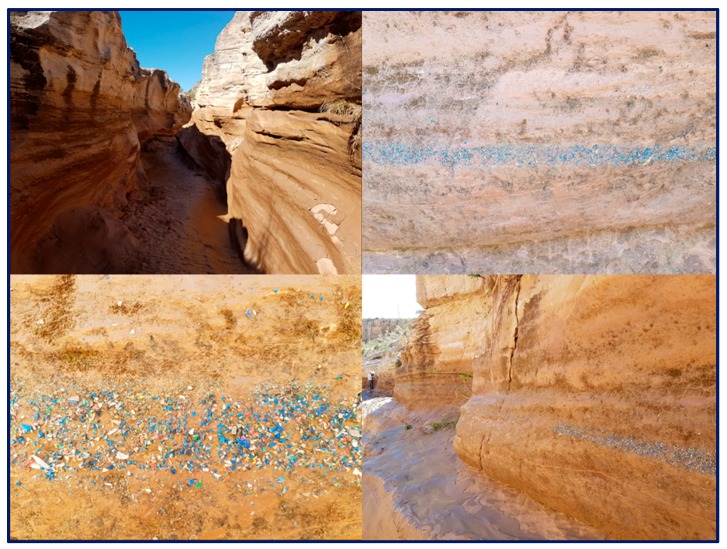
Appearance of the deposition and stratification of plastic materials in a Spanish canyon (Source: [16]).

Figure 1 shows a famous picture taken by Spanish students during a university trip. In the photo, the flood level river in the canyon bed is well-recorded thanks to the deposition of plastic micro-fragments that by now have been well-mixed with the sedimentary curly making up the canyon.

**Figure 2:**
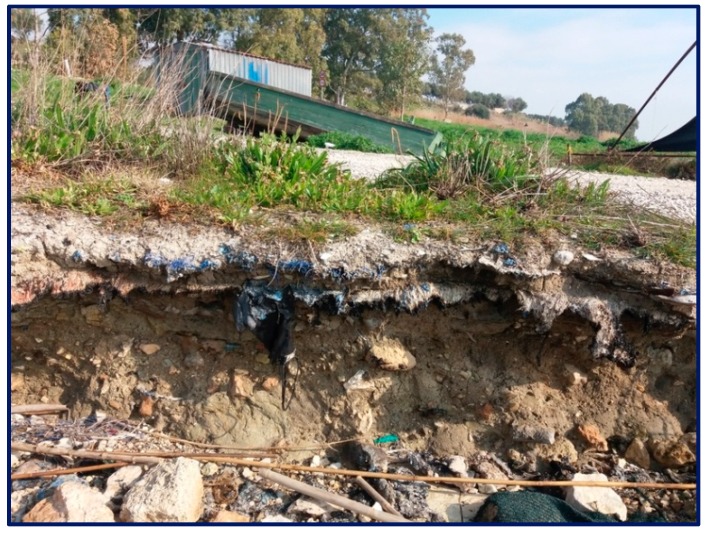
Layering of plastic materials in an area of Southern Italy.

**Figure 3:**
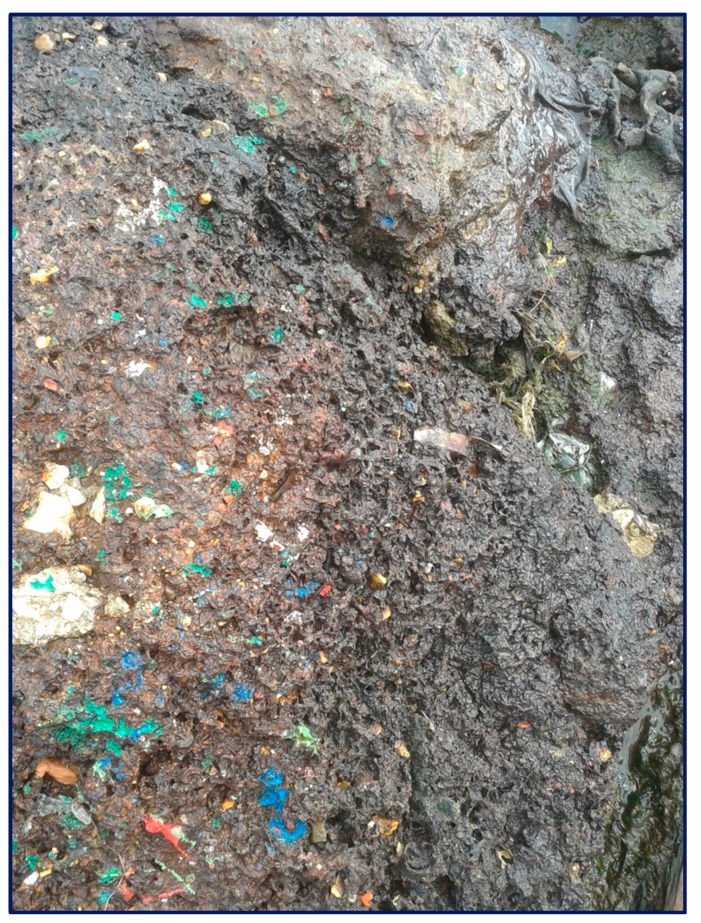
Detail of the plastic stratigraphy.

We found a similar situation in Southern Italy; indeed, in Figure 2 and Figure 3, it is possible to observe that plastics were even used to fill the road surface, probably to obtain a double advantage: no disposal costs for materials and no costs for the use of suitable materials (excavated rocks).

According to the layers, once accumulated and stratified, the sediment, which consists of fragments of various plastic sizes, can have a good conservation potential that is comparable to that one of recalcitrant organic fossils. Such synthetic fossil-based materials are so abundant and widespread on Earth that we can consider them “technofossils” as they will constitute a perennial proof of the existence of humans on Earth [4] to the point of being able to define this historical epoch as the Plasticene [14,15].

## 2. Plastics and Co-Contaminants

Microplastics (MPs) are defined by [17] as “synthetic solid particles or polymeric matrices, with regular or irregular shape and with size ranging from 1 μm to 5 mm, of either primary or secondary manufacturing origin, which are insoluble in water.”.

A key concern of microplastics pollution is whether they represent a risk to ecosystems and human health. However, there is much uncertainty associated with this issue. Data on the exposure and effect levels of microplastics are therefore required to evaluate the risk of microplastics to environments and human health. The adverse effects on organisms that are exposed to microplastics can be separated into two categories: physical effects and chemical effects. The former is related to the particle size, shape, and concentration of microplastics, and the latter is related to hazardous chemicals that are associated with microplastics. Though data on microplastic exposure levels in environments and organisms have rapidly increased in recent decades, limited information is available on the chemicals that are associated with microplastics.

Microplastics can contain two types of chemicals: (i) additives and polymeric raw materials (e.g., monomers or oligomers) originating from the plastics, and (ii) chemicals absorbed from the surrounding ambience.

Additives are chemicals intentionally added during plastic production to give plastic qualities like color and transparency and to enhance the performance of plastic products to improve both the resistance to degradation by ozone, temperature, light radiation, mold, bacteria and humidity, and mechanical, thermal and electrical resistance [18].

They include inert or reinforcing fillers, plasticizers, antioxidants, UV stabilizers, lubricants, dyes and flame-retardants [18].

Among the charges, wood and rock flour, clay, kaolin, graphite, glass fibers, cotton flakes, jute or linen, cellulose pulp, etc. are used [18]. According to the definitions proposed by the American Society for Testing and Materials (ASTM-D-883), inert fillers are materials that are used to modify the strength, working and flow properties, and shrinkage of plastics, while the reinforcing ones, also called fillers, are defined as those with some strength properties that are significantly superior to those of the base resin [19]. These fillers (such as carbon black in rubber), which are mixed in with the polymer, result in an interface volume that is generated at the filler-resin contact surface. It is the superior properties of this interface layer that obtain increased modulus and mechanical properties such as impact strength or tensile strength in the composite polymer. As the effect is surface-related, the smaller particle sizes of fillers generally yield a better reinforcing effect. There are clays, silica, glass, chalk, talc, asbestos, alumina, rutile, carbon black, and carbon nanotubes [20].

Plasticizers are complex chemical products that have low vapor pressure, are insoluble in liquids, are chemically stable, and which are inserted between molecular chains to reduce their forces of physical attraction and increase their mobility, workability or distensibility. In this way, the flexibility and plasticity of a resin that is processed and the impact resistance of the product during use are increased [21].

Because plastics are particularly sensitive to the degrading action of light, UV radiation and heat, the stabilizers, have the function of preventing the thermal decomposition during the processing, as well as the oxidation and the consequent breaking of the polymeric chains (using phenols and aromatic amines). They mainly consist of organic or inorganic cadmium, barium, or lead salts [22].

Soluble or insoluble dyes are organic or inorganic substances in the form of fine powders that give the polymer the desired color; the soluble dyes maintain the transparency of the plastic, while the insoluble ones (pigments) cover it to make it opaque. Many inorganic pigments contain heavy metals, while organic pigments include various chromophoric families like azo pigments, phthalocyanine pigments, anthraquinone chromophores, and various other chromophores [23].

Lubricants and anti-adhesives are substances that facilitate the processing of plastic materials, improving their flow characteristics. They consist of calcium or magnesium stearates [24].

Flame retardants have the function of cooling or protecting a material in the event of a fire by preventing the oxidation of flammable gases or by forming a layer of ash. They are products that contain, for example, chlorine and bromine, which release by the action of the flame; phosphorus, which favours the transformation into coal; and aluminium hydroxide, which generates water vapour and CO_2_ at 200 °C [24].

The additives, in almost all cases, are not chemically bound to the plastic polymer; only some flame retardants are polymerized with plastic molecules, becoming part of the polymeric chain [18].

Though these additives improve the properties of polymeric products, many of them are toxic, and their potential for the contamination of soil, air and water is high [18]. Studies on their impact on aquatic organisms with which they come into contact through macro and microplastics ingestion are still ongoing [25,26].

The combination of various kind of polymers of different sizes and shapes that are joined to the action of a large amount of additives that originate from plastics results in a cocktail of contaminants that not only alter the nature of plastic but can leach into the air, water, food, and, potentially, human body tissue during their use or their disposal, thus exposing us to several chemicals together.

### 2.1. Additives of Concern

Many substances that are classified as hazardous according to the EU regulation on classification and labelling [27] are present in everyday products as regular ingredients.

The toxicity of a substance is its ability to cause harmful effects. These effects can strike a single cell, a group of cells, an organ system, or the entire body. Chemicals that are considered most harmful are those that cause cancer, mutations to DNA, have toxic reproductive effects, are recalcitrant into the environment, are capable of building up in the food chain or bodies, and other harmful properties, such as disrupting hormones [28,29]. The internal organs that are most commonly affected are the liver, the kidneys, the heart, the nervous system (including the brain) and the reproductive system [29,30].

Among these chemicals, many routinely used to make plastics are dangerous. Bisphenol A (BPA), phthalates, as well as some of the brominated flame retardants, that are used to make household products and food packaging, have been proven to be endocrine disruptors that can damage human health if ingested or inhaled [30].

Endocrine-disrupting chemicals (EDCs), identified as substances that are exogenous to the human or animal organism, have hormonal activity that alters the homeostasis of the endocrine system, so they are of particular concern. These compounds interfere with the development of the endocrine system and affect the functioning of organs that respond to hormonal signals. The endocrinal and reproductive effects of endocrine disruptors may be a consequence of their ability to: (a) mimic natural hormones, (b) antagonize their action, (c) alter their pattern of synthesis and metabolism, or (d) modify the expressions of specific receptors [31,32,33].

Recent science has associated EDCs with various diseases and conditions, such as hormonal cancers (breast, prostate, testes), reproductive problems (genital malformations, infertility), metabolic disorders (diabetes, obesity), asthma, and neurodevelopmental conditions (learning disorders, autism spectrum disorders). Alongside the already shown scientific evidence, concern exists because of the rising levels of many diseases in Europe and worldwide. Additionally, the public is widely exposed to these chemicals from various sources [30].

#### 2.1.1. BPA

BPA is a carbon-based synthetic compound with formula C_15_H_16_O_2_ and a structure that contains two 4-hydroxyphenyl groups, which give to it a mild phenolic odor. It was first synthesized in the 1890s by the condensation of acetone with two equivalents of phenol [33].

BPA is a common plasticizer that is used in industry, especially in polycarbonate plastics manufacturing processes and food packaging [34,35].

BPA-based polycarbonate plastics are robust and stable because they can endure exposure to high temperatures and sustain high-impact collisions. These characteristics make them valuable as components of safety equipment and food packaging as they withstand heating in microwave ovens. Because it is a component of epoxy resins in protective coatings, such as the insides of aluminum and metal cans (as well as the lid closures of glass jars and bottles), BPA helps to extend the shelf life of food and beverage products [35,36]. Even if the compound is highly persistent, its instability within plastic products facilitates leaching, thus reporting a high prevalence in aquatic environments, particularly in landfill leachates [37,38].

In the early 1930s, Dodds and Lawson discovered that BPA was estrogenic [39], and, recently, the General Court of the EU confirmed that it is a ‘substance of serious concern’ for its hormonal disrupting properties on the human body. The Court upheld a previous decision by the European Chemicals Agency (ECHA) to identify the substances that are used in the manufacture of plastic products such as water bottles, food containers and receipts. It has been confirmed in several studies to be associated with obesity, cardiovascular disease, reproductive disorder, and breast cancer [30,40,41,42], and so it has gained increasing attention over the last decade, especially in terms of human safety. The contamination of food from BPA has been estimated to be responsible for 12,404 cases of childhood obesity and 33,863 cases of newly incident coronary heart disease in 2008. Another study estimated that BPA in food contact materials and thermal paper was likely responsible for 42,400 obese four-year-olds in Europe (with health costs of 1.54 billion euros per year) [30]. It is still under discussion if microplastics are relevant pollutant vectors for uptake into organisms in comparison to further uptake pathways, e.g., via water or sediment particles, even if studies regarding the level of bisphenol A adhered on microplastics surface are very limited.

The first study that investigated the presence of BPA on microplastics sampled from the remote, open ocean and urban beaches from America and Europe, reported concentrations ranging from 1 to 729.9 ng/g [42]. In most locations, including urban coasts, only trace concentrations (<1 ng/g) of BPA were detected. Due to its lower hydrophobicity (log n-Octanol/Water Partition Coefficient (Kow) = 3.40), the sorption of significant concentrations of BPA to marine plastics is unlikely. Indeed, in plastic fragments from remote coasts (730 ng/g) and open ocean fragments (283 ng/g), sporadic high concentrations of BPA were detected. Its utilization explains these higher BPA concentrations because it is a component of the plastic products and an additive. Indeed, BPA is a constituent monomer of polycarbonate plastic and epoxy resin and unreacted monomers in the plastics and resin and, degradation products from the polymers, can leach to the environment. Moreover, BPA is also used as additive to some plastics, and the leaching of BPA from commercial plastic products and dumped plastics can occur [42].

In the study of [43], the authors analyzed how the presence of non-suspended microplastics (polyamide particles (PA), which aggregated at the water surface or settled) modifies the acute effects of the environmental pollutant BPA on freshwater zooplankton (*Daphnia magna*). Daphnids are exposed to PA particles and BPA alone in a first step, and they are combined in a second step with a fixed concentration of PA and varying concentrations of BPA. All BPA concentrations used in the experiment greatly exceeded concentrations of the BPA that has been detected in rivers and lakes. The concentration of the PA particles used was also above the expected values in freshwater environments. There were two possible uptake pathways for BPA included in the experiments: direct uptake by BPA that was dissolved in water and vector-based uptake by the ingestion of PA particles that were loaded with BPA. The immobilization of daphnids was analyzed as an experimental endpoint to directly determine the influence of microplastics on pollutant toxicity. The results showed grazing by daphnids on settled PA particles from the bottom of the test beakers with high uptake rates that ensured the availability of PA particles, which could then potentially act as vectors for BPA. The analytical measurements showed that PA particles alone did not induce adverse effects, while the effects of BPA alone followed a typical dose-dependent manner. The sorption of BPA to PA particles before exposure led to a reduction of BPA in the aqueous phase. The combination of BPA and PA led to decreased immobilization, although the daphnids ingested PA particles that were loaded with BPA. These results showed the lower BPA body burden of daphnids in the presence of PA particles.

Another study [44] evaluated the retention of polyvinyl chloride (PVC) microplastics in sewage sludge during wastewater treatment. A model-based analysis indicated that PVC microplastics influenced the methane production from the anaerobic digestion of waste-activated sludge (WAS).

The presence of PVC microplastics (1-mm 20, 40, and 60 particles/g) inhibited methane production from WAS during anaerobic digestion to 90.6 ± 0.3%, 80.5 ± 0.1%, and 75.8 ± 0.2% of the control, respectively. Bisphenol A (BPA) leaching from PVC microplastics was the primary reason for the decreased methane production, causing significant (*p* = 0.037, 0.01, and 0.004) inhibitory effects on the hydrolysis–acidification process. The results of relevant enzyme activities also confirmed this.

#### 2.1.2. Phthalates

Phthalates are esters of phthalic acid (1,2-benzene dicarboxylic acid) on which there are two carbon chains of different lengths. Phthalates are a class of compounds that are produced in high quantities; they are the largest class of synthetic chemicals when considering production volume [45]. The authors of [46] reported that approximately 6,000,000 t/year phthalates are produced throughout the world. This production has remained quite constant for the past 20 years.

Their primary use is as plasticizers that are added to basic plastic material to impart specific qualities such as flexibility, pliability, and elasticity to plastic polymers [47].

They are colorless, odorless, oily liquids with low volatility and low water solubility [48]. Some phthalates have proven to be of concern due to their adverse effects to humans and ecosystems. Indeed, many phthalates are documented endocrine disruptors, and they are suspected of being endocrine disruptors, of affecting the reproduction of human beings, animals, or o being carcinogenic [49,50,51].

The problem is enhanced by the fact that several phthalates have similar modes of action, and that the overall risk, therefore, could increase when people and the environment are exposed to the different phthalates. Therefore, it is necessary to take the possible combination effects as a result of exposure to other phthalates and other substances into account [52]. There are many different types of phthalates, and there are indications that these do not have the same effects on the environment and human health.

Since 2007, there has been a ban in the EU on di(2-Ethylhexyl) phthalate (DEHP), dibutyl phthalate (DBP) and butyl-benzyl-phthalate (BBP) in all toys and childcare articles in concentrations above 0.1% (entry 51 of Annex XVII of the Regulation of the European Union (REACH) [53], as well as bans on diisononyl phthalate (DINP), diisodecyl phthalate (DIDP) and di-n-octyl phthalate (DNOP) in toys and childcare articles that can be placed in the mouth in concentrations above 0.1% [52,53]. DEHP is classified as reprotoxic category two and as T (toxic), while DBP is well-documented as having toxic effects on reproduction, as well as prenatal and postnatal development, in animals, and it is classified as reprotoxic category 3, as T (toxic), and as N (dangerous for the environment) [54]. DBP and diethyl phthalate (DEP) are the most widely used phthalates in medicinal products, even if toxicological effects have been observed in animals; as it cannot be ruled out that these findings have clinical relevance, the European Medicines Agency (EMA) is in the process of preparing limits for the use of DBP in medicines. Furthermore, the agency will probably also establish limits for the use of DEP and polyvinyl acetate phthalate (PVAP) in medicines [52].

Because of the potential risk of DEHP and DBP [55,56,57,58] and the potential hazard of the other phthalates, this group is considered as a hazard category [59].

With the publication of the new regulation [60] (EU) No. 2018/2005, which modifies Annex XVII of the REACH, the European Commission reinforces the limitations that are related to the presence of some phthalates in consumer products.

Limits on the presence of some phthalates were already present in the European legislation for the protection of consumers, but they were limited to “childcare articles,” that is “intended to reconcile the sleep or relaxation of children, their hygiene and their nutrients chin or sucking; “ these include teats, pacifiers, baby bottles, food containers and cutlery for children, and teethers for babies.

The new regulation extends the limitation to the following four phthalates (DEHP, DBP, BBP, and diisobutyl phthalate (DIBP)) also to other product and user groups. In particular, these four phthalates cannot be present in an amount higher than 0.1% of the plastic material (the limit applies to the sum, not only to the single phthalates) or in any article realized (in whole or in part) in a plasticized material—that is, in one or more of the following six materials:

- polyvinyl chloride (PVC),
- polyvinylidene chloride (PVDC),
- polyvinyl acetate (PVA),
- polyurethanes
- any other polymer (including polymeric foams and rubber) except silicone rubber and natural latex coatings;
- surface coatings, non-slip coatings, finishing products, decals, prints;
- adhesives, sealants, inks and paints.

The limitations provided by the regulation will come into force from 7 July 2020, but with some exceptions, as specified in Annex I of the regulation.

The consultation of the European Union rapid alert system for product safety (RAPEX) showed that in 14 years (from 2005 to 2018), 1591 cases of harmful phthalates were reported in various products (of which about the 89% of Chinese origin), mostly toys 94% [61]. In 2018 alone, there were 206 reported cases of toys that contained harmful phthalates [62].

If we then consider the cases that were related to food containers (detected by the Rapid Alert System for Food and Feed (RASFF), which is similar to RAPEX but reserved for the food sector), we find that from 2007 to 2018, there were as many as 108 cases of phthalate-contamination in food containers, an issue which came with the risk of ingestion through food products [63].

A study conducted in 2011 in Harbin and Shanghai (China) [64] analyzed the presence of nine phthalate esters in eight categories of foodstuffs. DEHP was the primary compound that was found in most of the food samples, with concentrations that ranged from below the limit of quantification (LOQ) to 762 ng/g wet weight.

Among the more frequently mentioned endocrine disruptors (EDCs), phthalates are of particular concern due to their ubiquity and to the higher levels found in the environment compared to other EDCs [65,66,67,68]. The detection of phthalates in purely domestic wastewater (the waste of plastic households) has also highlighted the leaching of phthalates from plastic during use into the environment [67,68,69].

Due to the high octanol–water partition coefficients, the strong sorption of phthalate esters (PAEs) by soil and sediment organic matters, biochar, and other carbonaceous sorbents has been reported [70,71]. However, the sorption behavior of PAEs on microplastics has not been studied systematically. Considering the hydrophobic surface of microplastics, they may have high sorption capacity for PAEs, which may pose a high environmental threat [72].

Recently, the authors of [73] investigated the sorption behavior of two PAEs, diethyl phthalate (DEP) and dibutyl phthalate (DBP), on three types of microplastics with particle sizes of less than 75 μm (PVC: polyvinyl chloride; PE: polyethene; and PS: polystyrene), and they demonstrated that hydrophobic interaction governed the partition mechanism. The sorption of the two PAEs on the three microplastics followed the order of PS > PE > PVC. For each kind of microplastics, the sorption of DBP was almost 100 times higher than that of DEP, demonstrating that the hydrophobic interaction dominated the partition. The results indicated too that the physical properties of microplastics did not play an essential role in their sorption behaviors. Moreover, on the one hand, solution pH (in the range of 2.0–7.0) and natural organic matter had no significant impact on the PAEs’ sorption by microplastics, thus indicating that microplastics could accumulate hazardous PAEs in different aquatic environments. On the other hand, the presence of NaCl (0–600 mM) and CaCl2 (0–300 mM) enhanced the sorption of both DEP and DBP on microplastics because of the salting-out effect.

The authors of [74] investigated organophosphorus esters (OPEs) and phthalic acid esters (PAEs) in beached microplastics that were collected from 28 coastal beaches of the Bohai and the Yellow Sea in north China. The analyzed microplastics included polyethene (PE) pellets and fragments, polypropylene (PP) flakes and fragments, and polystyrene (PS) foams. Tris-(2-chloroethyl)-phosphate (TCEP), tris (1-chloro-2-propyl) phosphate (TCPP), and di-(2-ethylhexyl) phthalate (DEHP) were the three most predominant compounds found. The maximum Σ4 OPEs concentration found was 84,595.9 ng/g^−1^, almost three orders of magnitude higher than the maximum Σ9 PAEs concentration observed. The PP flakes and PS foams contained the highest concentrations of the additives in contrast to the PE pellets, which contained the lowest concentrations. Moreover, the authors found that the spatial differences and compositional variation of the additives among the different microplastics suggested different origins and residence times in the coastal environment. These differences indicated that the characteristics of chemical additives might be a useful approach when tracing sources of microplastics in the environment.

#### 2.1.3. Heavy Metals

Heavy metals are natural elements that have a relatively high atomic mass and a rather high density compared to water. Commonly, a density of at least 5 g/cm^−3^ defines a heavy metal and differentiates it from other “light” metals. Other, broader definitions for “heavy metals” require an atomic mass higher than 23 or an atomic number exceeding 20 [75,76,77]. However, these definitions are confusing and misleading due to the fact that they cause the inclusion of non-metals.

Therefore, some authors [78] have suggested that is better define “heavy metals” when referring to (1) transition elements; (2) rare earth elements, which can be subdivided into the lanthanides and the actinides, including La and Ac themselves; and (3) a heterogeneous group including the metal Bi, the elements that form amphoteric oxides (Al, Ga, In, Tl, Sn, Pb, Sb and Po), and the metalloids Ge, As and Te.

Even though heavy metals are naturally present in our environment (e.g., in the atmosphere, lithosphere, hydrosphere, and biosphere), their environmental contamination and their exposure to humans have mainly originated from various anthropogenic activities [77].

One of their primary uses is as additives in polymer products (e.g., colorants, flame-retardants, fillers, and stabilizers) (Table 1) during the production process to increase the properties of plastics.

Antimony oxide, aluminum oxide, and zinc borate are, for example, well-known flame retardants, as well as compounds that contain Cl and Br [18].

Metals such as Zn, Pb, Cr, Co, Cd and Ti are instead used as inorganic pigment-based colorants [22,79]; among these, colorants that contain cadmium and lead are used for all kinds of colored polymers, lending a coloration that goes from yellow to red. Chromium is mostly used for polymers such as PVC, polyethene and polypropylene, whereas cobalt acetate is used in blue paints, particularly in the production of bottles that are made of polyethene terephthalate.

Additionally, the presence of Ti in plastic products works as a TiO_2_ indicator that is used both as a white pigment and as a UV stabilizer [80,81,82].

As part of the additives category, the stabilizers are generally used to prevent plastic degradation due to high temperatures, UV radiation, oxygen, and other kinds of atmospheric agents in order to lengthen product life. Among them, we again find compounds based on lead and cadmium, antimony trioxide and compounds based on Sn, which are mostly used in the making of doors and windows made of polyvinyl chloride.

Finally, although synthetic polymers are usually resistant to microbial attacks, some microorganisms can use some additives as sources of energy in the presence of water. This phenomenon can be prevented by adding, during the production of the polymer, biocides such as As, Sb and Sn [22].

According to the United States Environmental Protection Agency (USEPA) and the International Agency for Research on Cancer (IARC), arsenic, cadmium, chromium, lead and mercury are classified as “known” or “probable” human carcinogens based on evidences of epidemiological and experimental studies that have shown a correlation between exposure to those elements and cancer incidence on humans and animals [83].

Their toxicity depends on many different factors like dosage, how the subject is exposed to the element, and chemical species, as well as age, sex, genetics and the nutritional state of the exposed subject. A high concentration of heavy metals causes cellular and tissue damage, leading to a variety of adverse effects and human diseases [84,85,86,87,88,89]. Among metals, Al, Sb, As, Ba, Cd, Cr (II), Co, Cu, Pb, Hg, Ni, Se, Sn and V are defined metal–estrogens showing high affinity to estrogen receptors because they can mimic estrogen activation; for this reason, they are considered harmful and potentially linked with breast cancer [89,90,91].

Cadmium has been suggested to take part in the promotion of cellular apoptosis and DNA methylation, in providing oxidative stress, in causing damage to DNA, in increasing bone fractures in postmenopausal women, and in lipid peroxidation [77,92,93].

Titanium oxide, for example, which is used as an additive in many plastics products, has been shown to generate cytotoxicity on human epithelial lung and colon cells [94]. Lead is responsible for a variety of consequences on human health such as affecting the DNA reparation system, producing ROS (reactive oxygen species), modifying the genes that are responsible for the cellular tumor regulation, and various effects on the central nervous system, including the damage of motor and cognitive functions, convulsions, coma, and death. Arsenic contamination could cause cancer to the urinary bladder, lungs, liver and kidneys. As for mercury, it affects two target organs: the central nervous system and the kidney. The toxicity of the elemental mercury is due to mercuric mercury. Inflated elemental mercury vapors promptly pass through the blood–brain barrier, and the consequent oxidation in mercuric mercury starts a connection with brain macromolecules [95].

The exposure of living organisms to such inorganic pollutants is ever increasing if we consider the interactions of microplastics, vectors themselves of metals, with biota [3,96].

Though polymers were considered to be inert towards metals in the past [97], great attention has recently been paid to better understanding the interaction between heavy metals and microplastics [98,99,100,101,102,103,104,105,106,107,108].

In this regard, earlies studies such as [98] investigated the ability of virgin and aged microplastics to adsorb metals. Plastic production pellets were collected from beaches and sediment flats of south-west England and revealed variable concentrations of trace metals (Cr, Co, Ni, Cu, Zn, Cd and Pb) that, in some cases, exceeded the concentrations that were reported for local estuarine sediments. The same authors studied the rates and mechanisms of metals that were associated with virgin and beached polyethene pellets in a laboratory-scale experiment. Trace metals were shown to adsorb to both virgin and beached pellets but with a higher rate on aged pellets. Presumably, metal adsorption proceeds through interactions between divalent cations (e.g., Cu^2+^, Cd^2+^, and Pb^2+^) and oxyanions (e.g., Cr_2_O_4_^2−^) with charged or polar regions of the plastic surface (effected by imperfections and the presence of charged contaminants and additives, for example), and via non-specific interactions between neutral metalorganic complexes and the hydrophobic surface of the bulk plastic medium. Aged beached pellets accumulate trace metals to a significantly greater extent, with equilibrium partition coefficients ranging from about 4 mL/g^−1^ (Co) to 220 mL/g^−1^ (Cr). Its reactivity is enhanced by changes to the polymer itself, as well as the presence of biofilms and chemical precipitates that enhance the critical role of plastic as a vehicle for the transport of metals in the marine environment.

In the study of [101], the authors examined, over the 14 days of the experiment, the adsorption of two heavy metals, copper (Cu) and zinc (Zn), that were leached from an antifouling paint to virgin polystyrene (PS) beads and aged polyvinyl chloride (PVC) fragments in seawater. They demonstrated that heavy metals were released from the antifouling paint to the water, and both microplastic types adsorbed the two heavy metals. The adsorption of Cu was significantly higher in PVC fragments than in PS, probably due to higher surface area and polarity of PVC. The concentrations of Cu and Zn increased significantly on PVC and PS throughout the experiment except for Zn on PS.

However, the absorption/desorption processes that can occur naturally in the environment are quite complex and present a high variability [109]. Indeed, several factors and variables can influence the interaction between metals and microplastics, such as the alteration of the plastic surface exposed to atmospheric agents, the increased roughness of aged particles compared to virgin materials, and the faster decomposition of darker particles [5]. All these components accelerate the degradation processes of microplastics, creating anionic and active sites that increase the interaction of particles with heavy metals [110].

Other significant variables to be considered responsible for increasing the interaction between microplastics and inorganic pollutants are related to pH, salinity variations, photo-oxidative erosion, the formation of biogenic biofilm, enhanced polymer polarity and plastic porosity [98,99,109,111,112].

The authors of [111] observed the Pb absorption capacity on nanoplastics (particles that are unintentionally produced within the size range from 1 to 1000 nm [113]) that were produced from microplastics that were collected on a beach exposed to the North Atlantic Gyre. Lead (II) adsorption kinetics, isotherm, and pH-edge analyses were carried out. The sorption reached a steady-state after around 200 min. The maximum sorption capacity varied between 97% and 78.5% for both tested Pb concentrations. Chemical reactions controlled lead (II) adsorption kinetics with the nanoplastics surface and to a lesser extent by intraparticle diffusion. Adsorption isotherm modelling demonstrated that nanoplastics were strong adsorbents that were equivalent to hydrous ferric oxides such as ferrihydrite. The adsorption was dependent on pH in response to the Pb(II) adsorption by the oxygenated binding sites that were developed on the account of the surface UV oxidation under environmental conditions. They could be able to compete with Fe or humic colloids for Pb binding, due to their amounts and specific areas, becoming efficient vectors of Pb and probably of many other metals.

Therefore, microplastics, once spread into the environment, with their load of intrinsic (additives) and extrinsic (environmental) heavy metals, can be conveyed into the food web to reach aquatic organisms [114,115,116,117,118,119] and then humans [120,121,122].

In this regard, the study of [123] pointed out the potential ability of metals that are present on marine microplastics in determining the co-selection of antibiotic-resistant human pathogens, representing a severe threat to humans that are exposed to the marine environment or even to seafood. Metals such as mercury, lead, zinc, copper and cadmium are accumulating to critical concentration in the environment and triggering the co-selection of antibiotic resistance in bacteria. In the marine environment, persistent pollutants like microplastics are recognized as a vector for the proliferation of metal/antibiotics, and human pathogens and horizontal gene transference between the phylogenetically distinct microbes that are present on microplastics are much faster than free-living microbes. Therefore, microplastics are an emerging global health threat [112].

However, studies on the impact of microplastics on human health are all in the early stages and need to be further developed [117,124].

#### 2.1.4. Flame-Retardants

**Figure 4:**
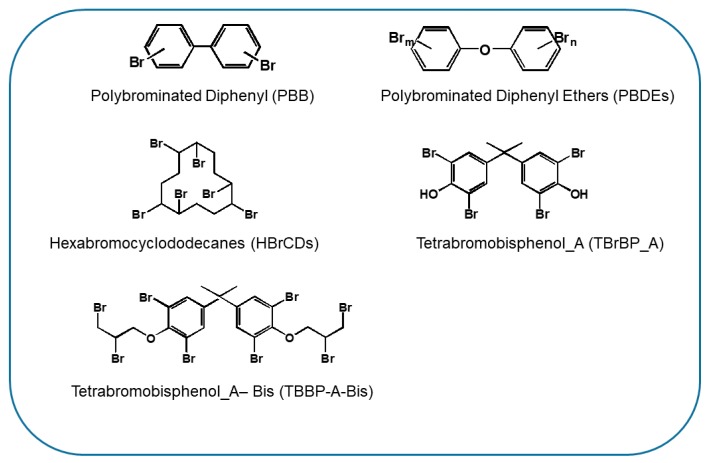
Chemical structure of some classes of halogenated flame retardants.

Flame retardants (FRs) are chemical compounds (Figure 4) that are capable of raising the flashpoint of the materials in which they are added. The main function of these molecules is, therefore, to prevent fires [125]. Flame retardants are divided into reactive and additive flame retardants according to their use. On the one hand, reactive chemicals are covalently bonded to polymers and are therefore less likely to reach the environment until the product is decomposed or burnt. The additive compounds, on the other hand, are only mixed with or dissolved in the material and can more easily migrate out of the product. Recently, over one hundred and forty types of flame retardants were counted, of which approximately seventy were found to belong to the brominated flame retardant (BFR) category. A first classification can be made based on their chemical nature—that is, organic or inorganic flame retardants:

- **Inorganic Flame Retardants:**
  Antimony Trioxide
  Aluminum Hydroxide
- **Organic Flame Retardants:**
  Tris (2,3-dibromopropyl) phosphate
  Short-chain chlorinated paraffin (10–13 carbon atoms) (SCCPs)
  Medium-chain chlorinated paraffin (14–17 carbon atoms) (MCCPs)
  Long-chain chlorinated paraffin (> 18 carbon atoms) (LCCPs)
  Polybrominated diphenyl (PBB)
  Polybrominated diphenyl Ethers (PBDEs)
  Hexabromocyclododecanes (HBrCDs)
  Tetrabromobisphenol_A (TBrBP_A)

Inorganic flame retardants act through different chemical and physical mechanisms [126]. With the application of heat, they can release water, they can release fire retardant gases that suffocate the flames, or, in other cases, they can form a protective film that protects the material in which they are inserted. Most often, inorganic flame retardants are used as adjuvants of organic ones. This is the case of antimony trioxide, which is used together with brominated flame retardants and acts as a catalyst in the decomposition reactions of BFRs [127]. Phosphorus-based flame retardants act in the solid phase [128]. With the application of heat, they form a polymer of phosphoric acid that carbonizes the material, blocking the pyrolysis process [128]. Though with some difference, the mechanism of action of organic flame retardants is the same. With the application of heat, they decompose even before the matrix that contains them, thus preventing the formation of flammable gases. In more detail, the halogens that are released by said molecules can react with the radicals H and OH, removing them from the chain reactions that are triggered during the combustion processes [129]. The critical factor that determines the goodness of the preventive action of these additives is their thermal stability, within the material that hosts them. If a retardant decomposes or evaporates too above or below the combustion temperature of the host material, its action will be ineffective. The brominated flame retardants decompose at a temperature of approximately 50 °C below the combustion temperature of the matrices to which they are inserted, therefore making them particularly useful for fire prevention [130].

**Figure 5:**
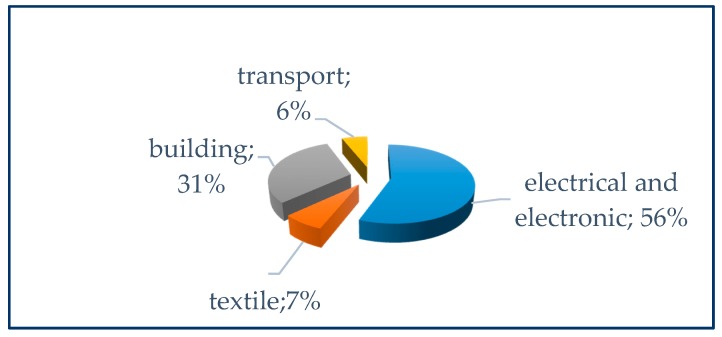
Greater fields of use of brominated flame retardants (BFRs) (Source data: [135]).

The use of chemical additives to make materials fireproof is not a recent phenomenon. The ancient Egyptians used hydrated potassium aluminum sulphate (KAl(SO_4_)_2_12H_2_O) to treat wood [131]. Following, Gay Lussac described a technique to protect theatre fabrics from fire through treatments with mixes of ammonium phosphate, ammonium chloride, and borax [132]. Today, the main fields of use of flame retardants concern the production of electrical materials, electronic materials, construction, textiles and transport (Figure 5). The massive growth in the production of plastic polymers has led to a substantial increase in the production of flame retardants. For example, in 1965, only 10% of bromine was used for the production of brominated flame retardants; this percentage became 40% in 1996 [133]. The global production of BFRs (as in the sum of Europe, Asia and the United States) increased from 106,000 metric tons in 1989 to 2,035,000 metric tons in 1999 [134].

The high concentrations of FRs that are found in plastic products are because these molecules (distinctly lipophilic) not only adsorb onto the surface of plastics and microplastics but also are present inside them because they are added as additives during the plastic production process [135].

Given the chemical inertia and marked lipophilicity of flame retardants, it is easy to intuit their rapid bioaccumulation. Different concentrations of PBDEs have been detected in various matrices including human milk, article glaciers, domestic dust, and, obviously, in sludge that is derived from water purification plants [136]. The considerable accumulation in this sludge deserves special attention if one thinks of the common practice of reusing it as an organic soil improver in agriculture. Another widespread practice of sludge disposal is its incineration. Several authors have already shown that the incomplete combustion of PBDEs leads to the formation of highly toxic species such as polybromodibenzofuranes (PBDF) and polybromodibenzodioxins (PBDD) [137]. In this regard, BFRs have been classified as “persisting organic pollutants” (POPs). The risk assessment made by the European community has shown that some of these molecules are toxic, suspected to be carcinogenic, and actively act on the endocrine system (endocrine disruptor).

Regarding PBDEs, the European community has banned the use of pentaBDE and octaBDE. This ban is because they are classified as toxic for reproduction [138]. DecaBDE will not be classified as a dangerous substance according to the European Directive 67/548/EEC because it is not toxic to human health or the environment. Several papers [139] have highlighted the immunotoxic effect of tetrabromobisphenol A (TBBP_A). Regarding HBrCDs, there is evidence [140] that they interfere with thyroid hormones.

Just like many other hydrophobic contaminants, evidence of attachment of PBDEs onto microplastics from marine environment has been highlighted in recent years [42,141,142], and the assimilation of these pollutants by organisms that ingest microplastics is highly probable [141,143,144]. Different levels of PBDE concentrations on microplastic samples have been observed based on polymer type and local anthropogenic activities. For example, the study of [42] underlined the presence of a much higher concentration of total-PBDEs that were analyzed on PP microplastics (9909 ng/g) than on PE samples (0.3 ng/g), with the most significant values being reported in open seas areas compared to remote locations. BDE-209 is one common PBDE congener that usually occurs in very high concentrations, and its low diffusion coefficient in LDPE implies a consideration when taking into account the risk that is posed by microplastic particle ingestion by marine organisms [42].

Indeed, amphipods have demonstrated the ability to assimilate PBDEs that are derived from microplastics and have shown a greater uptake for higher-brominated congeners (BDE-154 and -153 compared to BDE-28 and -47) [143]. In the cited study, amphipods (*Allorchestes compressa*) that were exposed to microplastics that were isolated from a commercial facial cleansing soap ingested ≤45 particles per animal and evacuated them within 36 h. Amphipods were exposed to polybrominated diphenyl ether (PBDEs) congeners (BDE-28, -47, -99, -100, -153, -154, and -183) in the presence or absence of microplastics. The results demonstrated that PBDEs that were derived from microplastics could be assimilated into the tissue of a marine amphipod. Microplastics reduced PBDE uptake compared to controls, but they caused a greater proportional uptake of higher-brominated congeners such as BDE-154 and -153, as compared to BDE-28 and -47. The study demonstrated that microplastics could transfer PBDEs into a marine organism by acting as a vector for the assimilation of POPs into marine organisms; thus, they pose a risk of contaminating aquatic food chains.

Another study [145] analyzed the feed of a typical commercial fish, the seabass, based on their absorption of microplastic-containing contaminants (PCBs and PBDEs). The study investigated how combinations of halogenated contaminants and microplastics that are associated with feed can alter toxicokinetics in European seabass and therefore affect the fish. Microplastic particles (2%) were added to the feed either with sorbed contaminants or as a mixture of clean microplastics and chemical contaminants, and they were then compared to a feed that contained contaminants without microplastics. For the contaminated microplastic diet, the accumulation of polychlorinated biphenyls (PCBs) and brominated flame retardants (BFRs) in fish were significantly higher, increasing up to 40 days of accumulation and then reversing to values that were comparable to the other diets at the end of accumulation.

The significant gene expression results of the liver (cyp1a, il1β, and gstα) after 40 days of exposure indicated that microplastics might indeed worsen the toxic effects (liver metabolism, immune system, oxidative stress) of some chemical contaminants that are sorbed to microplastics.

Moreover, on the one hand, at the end of the accumulation period, microplastics increased the bioavailability of the sorbed contaminants, showing a quadratic accumulation of all the 12 contaminants that were present on the microplastics. On the other hand, the metabolism of BDE99 to BDE47 (by debromination) in seabass was rather fast, and unlike other pollutants, this metabolism was unaffected by the presence of microplastics.

## 3. Effects of Micro and Nanoplastics on Human Health

A recent report from the “World Health Organization” [146] emphasized the ubiquitous microplastics presence in the environment and aroused great concern regarding the exposition and effects of nano and microplastics on human health [122,147,148,149,150]. One of the major nano and microplastic entry points into the human system is represented by the ingestion of contaminated food [8,151,152,153]. In a recent study conducted by [154], 0.44 MPs/g of nano and microplastics were found in sugar, 0.11 MPs/g were found in salt, 0.03 MPs/g were found in alcohol, and 0.09 MPs/g were found in bottled water. Humans could also assume an estimated intake of 80 g per day of microplastics via plants (fruits and vegetable) that accumulate MPs through uptake from polluted soil [155].

The presence of microplastics in marine species for human consumption (fish, bivalves and crustaceans) is now well-known [156]. As an example, in *Mytilus edulis* and *Mytilus galloprovincialis* of five European countries, the microplastic number has been found to fluctuate from 3 to 5 fibers per 10 g of mussels [116].

Therefore, following exposure via diet, uptake in humans is plausible, as evidenced by the capacity for synthetic particles smaller than 150 μm to cross the gastrointestinal epithelium in mammalian bodies, which causes systemic exposure. However, scientists speculate that only 0.3% of these particles are expected to be absorbed, while a lower fraction (0.1%) that contains particles that are bigger than 10 µm should be capable of reaching both organs and cellular membranes and passing through the blood–brain barrier and placenta [117]. Exposure concentrations are predicted to be low, although data about micro and nanoplastics into the environment are still limited due to the analytical and technical complications to extract, characterize, and quantify them from environmental matrices [157].

**Figure 6:**
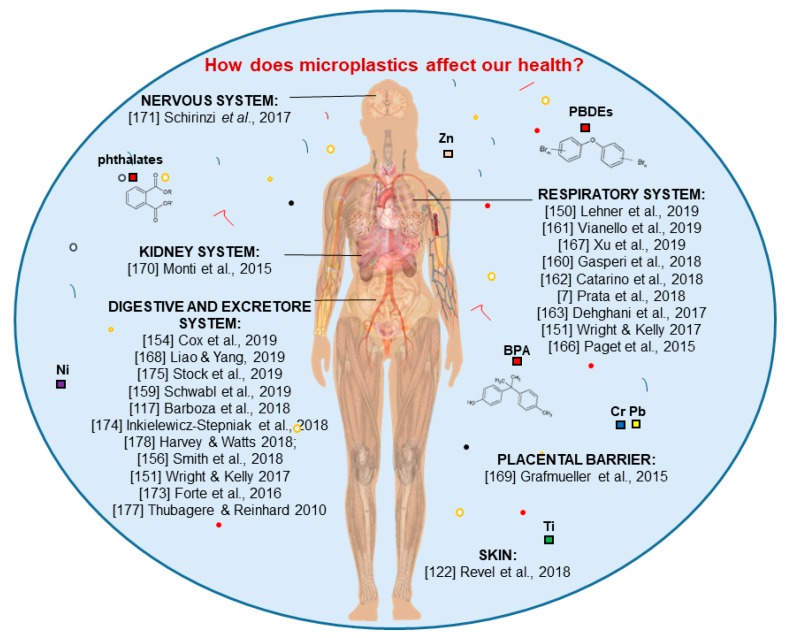
Overview of scientific studies focused on the effects of micro and nanoplastics on human health. Colored squares represent pollutants (organic and inorganic) that could be present in environmental matrices (free or associated with micro and nanoplastics) and that could enter into the human body through different entry routes.

Once ingested, particles smaller than 2.5 μm can enter the gastrointestinal tract (Figure 6) through endocytosis by M cells (specialized epithelial cells of the mucosa-associated lymphoid tissues) of Peyer’s patches. M cells transport particles from the intestinal lumen to the mucosal lymphoid tissues or through the paracellular persorption. Persorption consists of mechanical kneading of solid particles through gaps that are located in the single-layer epithelium at the villus tips of the gastrointestinal tract (desquamation zones) and into the circulatory system. The resulting toxicity is via inflammation due to the persistent nature of microplastics, as well as their unique properties such as hydrophobicity and chemical composition, and it is supposed to have an accumulative effect that is dependent on dose [151]. This assumption, regarding levels of microplastics in men at a gastro-intestinal level, was further confirmed by the finding of microplastics into human stools: Twenty plastic particles, mostly PE and PP (ranging in size between 5 and 500 mm), were found for every 10 g of stool [158,159]. Indeed, the human excretory system should be responsible for removing up to 90% of micro and nanoplastics ingested [156].

Another microplastics entry point to the human body is the aerial one (Figure 6) through inhalation [160,161]. The authors of [162] showed how the ingestion of synthetic fibers from mussel consumption is less than that of the ones that are inhaled from domestic dust during the same meal. The authors of [151] reported finding 18 fibers and four fragments/L of rain during precipitation events. Microplastics are carried by the wind or from atmospheric depositions and could also result from the erosion of agricultural and fertilized lands, dried sludges, and products from wastewater treatment, synthetic clothes fabric, industrial emissions, road-dust, marine aerosol. This spread could lead to respiratory distress, cytotoxic and inflammatory effects, and autoimmune diseases in men [7,128,131,135,163,164,165]. Moreover, the human lung has a quite wide alveolation surface of ca. 150 m^2^, with a very thin tissue barrier that is smaller than 1 μm and which could allow nanoparticles to penetrate the bloodstream and all human body [150]. Polystyrene particles of the size 50 nm have led to genotoxic and cytotoxic effects on pulmonary epithelial cells and macrophages (Calu-3 and THP-1) [166]. More widely, the response to inhaled particles, depending on differences on individual metabolism and susceptibility, may be expressed as immediate bronchial reactions (asthma-like), diffuse interstitial fibrosis and granulomas with fiber inclusions (extrinsic allergic alveolitis, chronic pneumonia), inflammatory and fibrotic changes in the bronchial and peribronchial tissue (chronic bronchitis), and interalveolar septa lesions (pneumothorax) [7]. For example, similar effects have been registered in workers of the textile industry in close contact to nylon, polyester, polyolefin and acrylic fibers. The low deterioration of microfibers has been found in patients suffering from pulmonary cancer as a confirmation of the bio-persistence of these synthetic particles. In addition to bio-persistence, fiber size has an impact in their toxicity [151]; for example, fibers of 15-20 μm cannot be successfully removed from macrophages to the lungs. Additionally, in [167], the toxicity of smaller-sized polystyrene nanoparticles (25 nm in diameter), which induced lower cell viability, cell cycle arrest in the S phase, the transcription of the activated inflammatory gene, and changed protein expression that was associated with the cell cycle and pro-apoptosis, was demonstrated. Not to be overlooked is the potential transmission of microorganisms through the microplastics that are present in the air. By attaching to microplastic surfaces in order to be protected from UV radiations, microorganisms could reach the lung and become another threat of infections to human health [7].

The last exposure pathway of microplastics to the human body could be skin contact (Figure 6) through water while washing or while using scrubs and cosmetics that contain micro and nanoplastics.

However, the penetration of the corneous layer is limited to particles lower than 100 nm, so it is unlikely that microplastics absorption could occur through the skin; on the contrary, nanoplastics absorption is more probable [122].

Though plastic is considered an inert material, there is a broad range of properties that characterize microplastics, such as size, shape, chemical composition, and hydrophobicity, that could cause harm and influence the cytotoxicity of particles to cells and tissues [151].

The increased surface area/volume ratio of microplastics, combined with their hydrophobicity, translates to a high affinity to a broad range of hydrophobic and persistent organic pollutants, antibiotics, and heavy metals that could be introduced in the human body by microplastics uptake.

In regard to heavy metals, an in-vitro study was conducted about chromium (Cr) absorption/desorption behavior in the human digestive system considering non-degradable MP types (polyethene (PE), polypropylene (PP), polyvinylchloride (PVC), and polystyrene (PS)) and degradable MPs (polylactic, PLA). The results showed the ability to release Cr (VI) and Cr (III) from MPs into the digestive-gastric phase thanks to stomach acid conditions that stimulated the process [168].

The interactions between microplastics/nanoplastics and other human organs are still being tested, but their possible effects can be assessed based on human absorption models of nanomaterials that are produced by various industrial production processes. In the studies of [169,170], the ability of nanoparticles in polystyrene to cross the placental barrier and the primary human renal cortical epithelial (HRCE) cells was demonstrated.

The use of metal nanoparticles (NPs) (AgNP and AuNP, ZrO_2_NPs, CeO_2_NPs, TiO_2_NPs, and Al_2_O_3_NPs), carbon nanomaterials (C60 fullerene, graphene) and polyethene (PE) and polystyrene (PS) microplastics has demonstrated that cytotoxic effects are induced on T98G and HeLa cell lines (human brain and epithelial cells) [171]. Additionally, the use of polypropylene (PP) particles has shown different but harmful effects on various cell lines, based on the size (~20 μm and 25–200 μm) and the different concentrations used in the various tests. Therefore, the interaction of microplastics with humans can produce cytotoxicity, hypersensitivity, unwanted immune responses, and acute responses like hemolysis, thus representing a potential risk to human health [172].

Recent in-vitro studies about effects of plastics on the human body have mostly used engineered nanoplastics that can influence their absorption and also the translocation and production of ROS due to their dimension, charge and shape [148,150,173,174,175,176]. In fact, in the study [174], the interaction between positively-charged nanoparticles of polystyrene (60 nm) and the secretion film of the gastrointestinal epithelium (first physical barrier after digestion) was analyzed. Nanoplastics showed a strong ability to interact with the secretion film, to influence cellular vitality, and to induce apoptosis in the intestinal epithelial cell lines LS174T, HT-29 and Caco-2. Those cytotoxic effects were already observed in the study of [177], which was carried out on adenocarcinoma colon–rectal human differentiated cells, Caco-2, by using polystyrene nanoparticles of 20 and 40 nm.

## 4. Conclusions

The intake of microplastics by humans is by now quite evident. The entry point may be through ingestion (through contaminated food or via trophic transfer), through inhalation, or through skin contact.

Following the intake of microplastics into the human body, their fate and effects are still controversial and not well known. Only microplastics smaller than 20 µm should be able to penetrate organs, and those with a size of about 10 µm should be able to access all organs, cross cell membranes, cross the blood–brain barrier, and enter the placenta, assuming that a distribution of particles in secondary tissues, such as the liver, muscles, and the brain is possible. Not enough information is available to fully understand the implications of microplastics for human health; however, effects may potentially be due to their physical properties (size, shape, and length), chemical properties (presence of additives and polymer type), concentration, or microbial biofilm growth.

How toxic chemicals adsorb/desorb onto/from microplastics is not well known, but plausible mechanisms include hydrophobic interactions, pH variations, the ageing of particles, and polymer composition. Furthermore, not enough studies have fully explained the primary sources of pollutants that are present on microplastics and whether their origin is extrinsic from the surrounding ambient space, intrinsic from the plastic itself, or, more probably, from a combination of both and from a continuous and dynamic process of absorption and desorption that is related to the spread of the particles into the environment and to their consequent exposure to weathering.

## Figures and Tables

**Table 1:** Main use of heavy metals as additives in polymer products and their effects on human health.

Heavy Metals	Additives	Type of Polymers	Effects on Human Health	References
**Antimony (Sb)**	Flame retardants and biocides	Various plastics	Metal–estrogen; breast cancer	[18,22,90]
**Aluminum (Al)**	Stabilizers, inorganic pigments and flame retardants.	PBT, PET, PE, PVC	Metal–estrogen; breast cancer	[18,22,90]
**Zinc (Zn)**	Heat stabilizers, flame retardants, anti-slip agents and inorganic pigments.	PVC, PE, PP	-	[18,22]
**Bromine (Br)**	Flame retardants	PBT, PE, PS, PP	Apoptosis and genotoxicity	[18,88]
**Cadmium (Cd)**	Heat stabilizers, UV stabilizers and inorganic pigments	PVC	Changes in metabolism of calcium, phosphorus and bone; osteomalacia and bone fractures in postmenopausal women; lipid peroxidation and in the promotion of carcinogenesis; cellular apoptosis; DNA methylation.	[18,22,77,79,92,93]
**Copper (Cu)**	Biocides	-	Formation of reactive oxygen species (ROS); inducing DNA strand breaks and oxidation.	[18,22,77]
**Mercury (Hg)**	Biocides	PU	Mutagen or carcinogen; induction of the disruption of DNA molecular structure and brain damage.	[11,22,77,95]
**Arsenic (As)**	Biocides	PVC, LDPE and polyesters	Congenital disabilities; Carcinogen: lung, skin, liver, bladder, kidneys; gastrointestinal damage; death.	[18,22,93]
**Tin (Sn)**	UV stabilizers and biocides	PU foam and PVC	Metal–estrogen; breast cancer; skin rashes; stomach complaints; nausea; vomiting, diarrhea; abdominal pain; headache and palpitations; potential clastogen.	[18,22,87,90]
**Lead (Pb)**	Heat stabilizers, UV stabilizers and inorganic pigments	PVC and all types of plastics, where red pigments are used	Anemia (less Hb); hypertension; miscarriages; disruption of nervous Systems; brain damage; infertility; oxidative stress and cell damage.	[18,22,77,79,90,93]
**Titanium (Ti)**	UV stabilizers and inorganic pigments	PVC	Cytotoxicity on human epithelial lung and colon cells.	[18,80,94]
**Cobalt (Co)**	Inorganic pigments	PET bottles	Formation of reactive oxygen species (ROS); neurological (e.g., hearing and visual impairment); cardiovascular and endocrine deficits.	[22,77,86]
**Chrome (Cr)**	Inorganic pigments	PVC, PE, PP	Allergic reactions to the body; nasal septum ulcer; severe cardiovascular, respiratory, hematological, gastrointestinal, renal, hepatic, and neurological effects and possibly death.	[77,79]
**Barium (Ba)**	Inorganic pigments and UV stabilizers	PVC	Metal–estrogen, breast cancer; cardiovascular and kidney diseases; metabolic, neurological, and mental disorders	[18,22,85,90]
**Manganese (Mn)**	Inorganic pigments	-	Neurodegenerative disorder	[18,84]
